# Rapid Discrimination of Clinically Important Pathogens Through Machine Learning Analysis of Surface Enhanced Raman Spectra

**DOI:** 10.3389/fmicb.2022.843417

**Published:** 2022-04-08

**Authors:** Jia-Wei Tang, Jia-Qi Li, Xiao-Cong Yin, Wen-Wen Xu, Ya-Cheng Pan, Qing-Hua Liu, Bing Gu, Xiao Zhang, Liang Wang

**Affiliations:** ^1^Department of Bioinformatics, School of Medical Informatics and Engineering, Xuzhou Medical University, Xuzhou, China; ^2^Department of Laboratory Medicine, School of Medical Technology, Xuzhou Medical University, Xuzhou, China; ^3^Department of Basic Medicine and Biological Science, Soochow University, Suzhou, China; ^4^State Key Laboratory of Quality Research in Chinese Medicines, Macau University of Science and Technology, Macao, Macau SAR, China; ^5^Laboratory Medicine, Guangdong Provincial People’s Hospital, Guangdong Academy of Medical Sciences, Guangzhou, China; ^6^Jiangsu Key Laboratory of New Drug Research and Clinical Pharmacy, School of Pharmacy, Xuzhou Medical University, Xuzhou, China

**Keywords:** surface enhanced Raman spectra, bacterial pathogen, machine learning, convolutional neural network, long short-term memory

## Abstract

With its low-cost, label-free and non-destructive features, Raman spectroscopy is becoming an attractive technique with high potential to discriminate the causative agent of bacterial infections and bacterial infections *per se*. However, it is challenging to achieve consistency and accuracy of Raman spectra from numerous bacterial species and phenotypes, which significantly hinders the practical application of the technique. In this study, we analyzed surfaced enhanced Raman spectra (SERS) through machine learning algorithms in order to discriminate bacterial pathogens quickly and accurately. Two unsupervised machine learning methods, K-means Clustering (K-Means) and Agglomerative Nesting (AGNES) were performed for clustering analysis. In addition, eight supervised machine learning methods were compared in terms of bacterial predictions *via* Raman spectra, which showed that convolutional neural network (CNN) achieved the best prediction accuracy (99.86%) with the highest area (0.9996) under receiver operating characteristic curve (ROC). In sum, machine learning methods can be potentially applied to classify and predict bacterial pathogens *via* Raman spectra at general level.

## Graphical Abstract

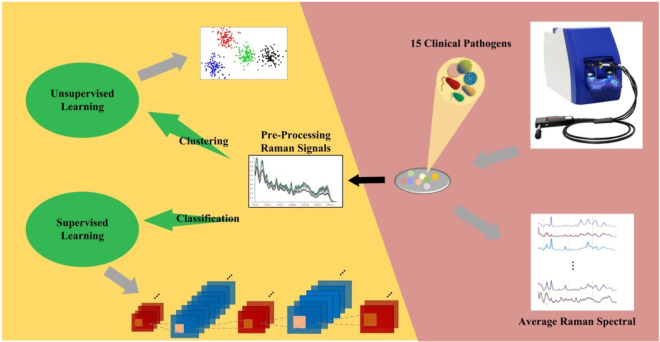

## Highlights

- Surface-enhanced Raman spectroscopy (SERS) has the potential to be used for detecting bacterial pathogens in clinical settings.
- Pretreatment of SERS spectra facilitates the increment of signal-to-noise ratio and increases the accuracy of bacterial detection rate.
- All machine learning algorithms showed their capacities in pathogen clustering and species discrimination via SERS spectra.
- Convolutional neural network showed highest accuracy and robustness in discriminating bacterial pathogens in terms of SERS spectral analysis.

## Introduction

Infectious diseases frequently cause major public health threats and risks due to long-standing, emerging, and re-emerging bacterial pathogens (Bloom and Cadarette, 2019), while rapid and accurate identification of the causing bacterial agents could greatly improve therapeutical effectiveness and reduce host mortality (Caliendo et al., 2013). Although conventional methods are reliable and accurate in clinical diagnosis of bacterial infections, they mainly rely on culture-based testing and biochemical analysis that yield results in days or up to weeks after sampling, not even mentioning the fastidious and viable but non-culturable (VBNC) bacterial species under laboratory conditions (Järvinen et al., 2009). Recently, matrix-assisted laser desorption-ionization time of flight mass spectrometry (MALDI-TOF MS) is emerging as an important tool in bacterial identification in clinical laboratories due to its rapidity, reliability, and cost-effectiveness (Singhal et al., 2015). However, MALDI-TOF MS also suffers disadvantages such as lack of a complete spectra database for known bacteria and inaccuracy of bacterial discrimination at genus, species and sub-species levels like *Shigella* and *Escherichia coli*, etc. (Sloan et al., 2017). Thus, advanced and diverse detection methods should be developed in order to facilitate the rapid and accurate diagnosis of bacterial infections in clinical settings.

Raman spectroscopy is a non-destructive chemical analysis based on interactions between the light and chemical bonds within a material, which could generate detailed fingerprinting spectra for a particular biological sample (Orlando et al., 2021). Cumulative studies show that Raman spectroscopy (RS) has the potential to rapidly analyze clinical samples and efficiently identify bacterial species in simple procedures (Ho et al., 2019). However, because of the intrinsically weak signal of Raman effect, surface enhanced Raman spectroscopy (SERS) has been developed for the analysis of biological samples, which not only greatly improves the detection capacity of bacterial pathogens but also opens new directions for the detection of analytes at very low concentrations (Kuhar et al., 2018). Due to the complexity of the raw Raman spectral data, traditional statistical methods are not sufficient for data analysis and pattern recognition (Wang et al., 2021), which hinders the application of Raman spectroscopy in the field of infectious diseases. With the assistance of advanced computational methods like machine learning methods, it would be possible for the promising technique to overcome current challenges and gradually realize its real-world applications in clinical laboratories for the detection of bacterial pathogens.

In this study, we analyzed a group of 15 bacterial pathogens belonging to different genera through SERS spectra. All the Raman spectra were processed to calculate average Raman spectrum for each bacterial genus, together with the corresponding characteristic peaks. In order to discriminate these bacteria efficiently, two representative unsupervised machine learning methods, K-means and agglomerative nesting (AGNES) were then applied to the spectral data for bacterial clustering. Moreover, three classic supervised machine learning methods, Random Forest (RF), Decision Tree (DT), and Support Vector Machine (SVM), together with five deep learning algorithms, Multilayer Perceptron (MLP), Convolutional Neural Network (CNN), Recurrent Neural Networks (RNN), Gate Recurrent Unit (GRU), and Long Short-term Memory (LSTM) were implemented to all the bacterial SERS spectra, the results of which were compared in terms of their prediction accuracies, sensitivities and specificities. In order to evaluate the robustness of supervised machine learning methods, artificial noise signals were added to the SERS spectra and the prediction accuracies were compared among algorithms, which revealed that CNN had the best capacity in terms of noise interference during species predictions. In sum, this study confirmed that Raman spectroscopy has the potential in clustering, discriminating and predicting bacterial pathogens from different bacterial genera in clinical settings with the assistance of machine learning algorithms.

## Materials and Methods

### Bacterial Strains and Chemical Materials

A total of 15 clinical bacterial pathogens studied in this experiment were directly isolated from clinical samples and cultured on Columbia blood agar (35°C, 18–24 h) at the Department of Laboratory Medicine, Affiliated Hospital of Xuzhou Medical University, which included six isolates of *Achromobacter xylosoxidans* (*n* = 610), nine isolates of *Burkholderia cepacian* (*n* = 600), 4 isolates of *Chryseobacterium indologenes* (*n* = 690), one isolate of *Corynebacterium glucuronolyticum* (*n* = 600), seven isolates of *Elizabethkingia meningoseptica* (*n* = 601), 20 isolates of *E. coli* (*n* = 38), five strains of *Micrococcus luteus* (*n* = 601), five isolates of *Moraxella catarrhalis* (*n* = 600), seven isolates of *Morganella morganii* (*n* = 130), five isolates of *Myroides odoratimimus* (*n* = 610), two isolates of *Neisseria flavescens* (*n* = 601), three isolates of *Providencia rettgeri* (*n* = 601), two isolates of *Pseudomonas putica* (*n* = 100), 18 isolates of *Serratia marcescens* (*n* = 569), and nine isolates of *Vibrio parahaemolyticus* (*n* = 600). The letter n represents the total number of SERS spectra for all the isolates of the same bacterial species. All bacterial pathogens were cultured, isolated, and then identified using MALDI-TOF MS and stored in freezer (Thermo Fisher Scientific, Waltham, MA, United States) at −80°C. Morphological, physiological, and clinical features of these bacterial pathogens were summarized in Supplementary Table 1. During analysis, all the strains were thawed, inoculated onto and cultivated on standard Columbia Blood Agar (CBA) for 24 h at 37°C. Single colonies were then randomly selected and mixed with negatively-charged silver nitrate nanoparticle (AgNO_3_) substrate for SERS study. For the preparation of the AgNO_3_ substrate, please refer to the procedures described by Tang et al. (2021).

### Surface-Enhanced Raman Spectroscopy

A single bacterial colony was mixed with 15 μL phosphate buffer saline (PBS) *via* vortexing, which was then mixed with 15 μL negatively-charged silver nanoparticle substrate solution. The mixed solution was placed onto silicon wafer and left on clean bench to air-dry completely. The commercial Raman spectrometer i-Raman^®^ Plus BWS 465-785H (B&W Tek, Plainsboro, United States) was used to measure the dried spot on the silicon wafer. Measurement parameters were set as following: excitation light source wavelength at 785 nm; laser power: 20 mW. Detection parameters were set as following: (1) spectrum acquisition: 5 seconds; (2) detector type: high quantum efficiency CCD array; (3) Raman shift range: 65-2800 cm^–1^; (4) resolution: less than <3.5 cm^–1^ at 912 nm. Finally, signals ranged from 519.56–1800.81 cm^–1^ were captured, which consisted of 657 value points in total for each spectrum.

### Average Raman Spectra and Characteristic Peaks

#### Removal of Outlier Raman Spectra

In this study, all the SERS spectral data for each clinical isolate were obtained from Raman spectrometer *via* the software BWSpec 4.02 (B&W Tek, United States). We identified outliers in the raw SERS spectra *via* variance contribution rate. The procedure was implemented through the PCount() function in the mvoutlier package of the R programming language (Filzmoser et al., 2008). Supplementary Table 2 showed the number of SERS spectra before and after outlier analysis.

#### Average Raman Spectra

By calculating the repeated Raman intensity of all samples under the same Raman shift for each bacterium, the average value of the intensity at the Raman shift was obtained, and then the average intensities at all the Raman shifts were calculated to generate the average Raman spectrum of the particular bacterial species. By following this procedure, 15 average Raman spectral curves were obtained, and the standard deviation (SD) of each average spectral line was calculated. Both average SERS spectra and 20% SD band were visualized by the origin software (OriginLab, United States). The width of the error band shows the reproducibility of Raman spectra for each bacterial species.

#### Identification of Characteristic Peaks

In order to discriminate the differences among different bacterial species through the SERS spectra, average Raman spectral curves was preprocessed through software LabSpec 6 (HORIBA Scientific, Japan), which included spectral smoothing, denoising, baseline correction, and normalization. After that, characteristic peaks in each average Raman spectrum were then identified. The specific parameter settings were *Degree* = 4, *Size* = 5 and *Height* = 50. The *smooth* function was used to denoise the spectrum. For the baseline correction, the parameters were set as *type* = Polynom, *Degree* = 6, *Attach* = No, and then the *Auto* function was applied to perform baseline fitting. Finally, the LabSpec software was used to fit the characteristic peaks. *GaussLoren* function was used with parameters set to *Level* = 13% and *Size* = 19 while other parameters were set by default. The normalization operation was performed for better comparing the curves of different bacterial species. The function *search* was used for the identification of characteristic peaks.

### Surface-Enhanced Raman Spectroscopy Spectral Preprocessing for Machine Learning Analysis

Before machine learning analyses, raw SERS spectra excluding outliers require a series of pre-processing steps, which includes spectral smoothing and denoising, baseline correction and normalization. Through preprocessing of SERS spectra, data quality was significantly improved and data dimensionalities were reduced, which greatly facilitated further statistical analysis of Raman spectra *via* supervised and unsupervised machine learning algorithms.

#### Smoothing and Denoising of Raman Spectra

Curve smoothing and denoising were performed to remove noise signals in SERS spectra caused by dark current and fluctuation of the external environment in order to improve the signal-to-noise ratio (SNR). There were a variety of filtering algorithms that could effectively reduce noise interference in the Raman spectra, which included moving window averaging and Savitzky-Golay filters and so on. In this study, Savitzky-Golay filtering method in the Unscrambler^®^ X software was used, which was a weighted average method highlighting the effect of the center point and calculating the filter value with a window. In particular, it is noteworthy that the number of points on both the left and right of the center point was set to three while the derivative order was set to two during data analysis procedures.

#### Baseline Correction and Spectral Normalization

Due to the noise interference in Raman spectroscopy, it was necessary to perform baseline correction on Raman spectra. Commonly used baseline correction methods include polynomial fitting based on least squares and asymmetric least squares (Baek et al., 2015). In this study, we used the *Baseline function* under *Transform* in the software Unscrambler^®^ X to perform baseline correction of previously smoothed SERS spectra. For parameter setting, *Rows* and *Cols* were set to *All*, and the *Method* was set to *Baseline offset*. In addition, each SERS spectrum in this study contains 657 Raman shifts. In order to remove the influences of signal intensities in the spectral data among different samples of the same species, max-min normalization by column for each spectrum was used.

### Machine Learning Analysis

#### Unsupervised Machine Learning

This study used two clustering algorithms to evaluate whether SERS spectral data belonging to 15 bacterial species were separable. SERS spectra were first pre-processed *via* removal of abnormal spectra, curve smoothing and denoising, baseline correction, and normalization as described above. Principal component analysis (PCA) was then applied to identify the principal components according to their cumulative contribution values, and only principal components with contribution value greater than 95% were retained. That is, the top m principal components whose cumulative variance contribution rate reached 95% were kept. Two clustering algorithms, K-means and AGNES were used to analyze the dimensionality-reduced SERS spectral data *via* Python *sklearn* package. The n_cluster parameter of the two clustering algorithms was preset to 15, and the linkage parameter of AGNES was set to *ward*, which minimized the sum of squared distances between all clusters.

#### Supervised Machine Learning

This study constructed and compared three classical supervised machine learning algorithms (RF, DT, SVM) and five deep learning algorithms (MLP, CNN, Simple RNN, GRU, LSTM) in terms of their capacities in predicting Raman spectra into different bacterial species, through which the best model(s) were identified. The pre-processed SERS spectra for each bacterial species were divided into training set, validation set and test set by following the ratio of 6:2:2 while the labels in the dataset were converted into the one-hot encoding form. In particular, one-hot encoding mainly uses N-bit state registers to encode N states. Each state has its own independent register bits, and only one bit is valid at any time. In simple terms, it is the representation of a categorical variable as a binary vector. Except for the integer index representing the variable, all other values are 0, while the variable is marked as 1.

**FIGURE 1:**
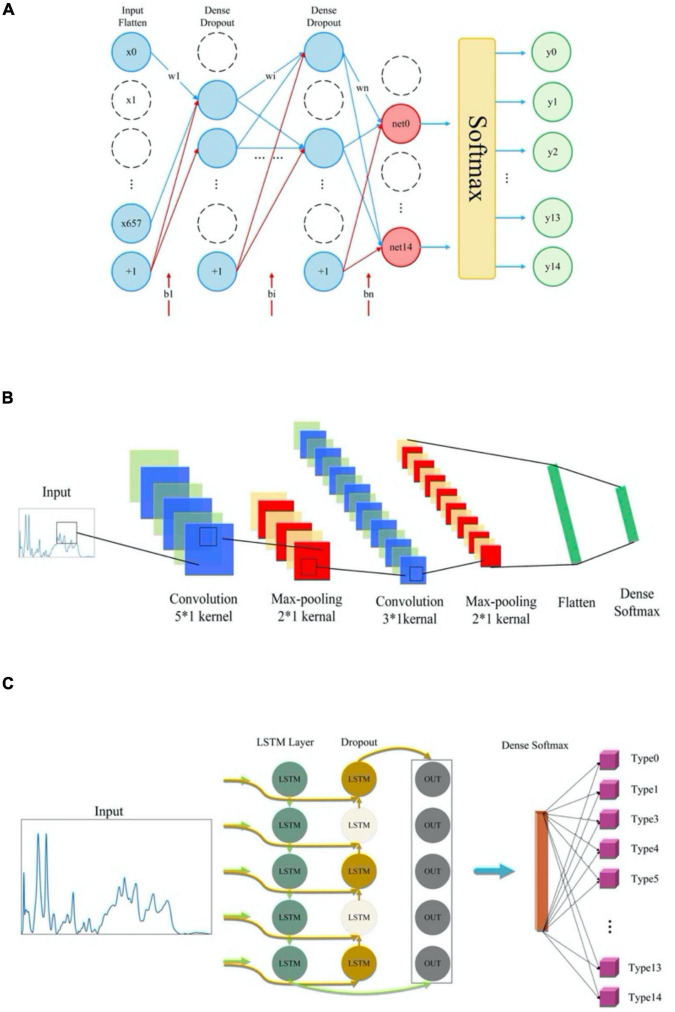
Schematic illustration of the network structures of deep learning algorithms. **(A)** Multi-layer perceptron model architecture. **(B)** Convolutional neural network (CNN) model architecture. **(C)** Network structure of three recurrent neural networks (RNN) models.

Deep learning methods included two models, CNN and RNN, in which MLP had a special CNN network structure while SimpleRNN and GRU were simplified LSTM model (Figure 1). In particular, CNN model consisted of one input layer, six convolutional layers with convolution kernel sizes of 5*1 and 3*1, three maximum pooling layers, one fully connected layer and a *softmax* output layer that achieved 15-dimensional outputs. As for MLP, its network structure consisted of one input layer, four fully connected layers and one output layer. The activation function was selected as *relu*. In specificity, the framework of MLP neural network model in this study included an input layer, three hidden layers and an output layer. Each hidden layer is paired with a dropout layer. The rate of dropout is 0.2 while the activation function of each hidden layer is used *relu*. For the output layer, units are set to 15, and the activation function selects *softmax* for multi-classification. Dropout layer was mainly used to prevent curve overfitting and enhance the generalization ability of the model. As for LSTM, SimpleRNN and GRU, the three RNN modes were composed of three RNN layers, two Dropout layers and a fully connected layer. All the machine learning models were fitted and trained on the training dataset, and their optimized parameters were listed in Supplementary Table 3.

Accuracy rates (ACC) were compared in terms of prediction abilities of different supervised machine learning algorithms on SERS spectra. In order to verify the reliability of ACC, 5-fold cross-validation was also performed, which could eliminate the adverse effects of unbalanced data divisions. Moreover, this study also used F1-score (F1) as an additional metric, which was equivalent to the harmonic average of Precision (Pre) and Recall. The larger F1 was, the better the model performance was. Similar to accuracy and recall rate, receiver operating characteristic (ROC) curve also functioned as a measurement of the model quality. The optimal machine learning model was then selected based on all the above-mentioned indicators. In order to assess how many bacterial genera were misjudged as other genera, this study also used a confusion matrix of CNN to quickly visualize the proportion of misclassified genera.

#### Robustness of Machine Learning Models

Addition of noises to existing Raman spectral data could evaluate the performance of different models in terms of their predicting capacities. In this study, we added artificial noise with different signal-to-noise ratios (SNRs) to the pre-processed data set in order to test model robustness. The specific process was as follows: set different values of SNR in advance (SNR = 1, 2, 3, 5, 15, 25, 35); generate the required input noise D randomly (Formula 1); calculate SNR through dividing the signal power (Power of Signal, PS, Formula 2) with noise power (Power of Nosie, PN, Formula 3); calculate the required noise ND (Formula 4) through PN and D; and the noisy signal (NS, Formula 5) was finally obtained.


(1) $$D=d_{\max}-\frac{\sum_{i=1}^{n}d_{i}}{n}\left(d_{i}\in \left(0,1\right)\right)$$

(2) $$PS=\sum {\left|x\right|}^{2}$$

(3) $$PN=\frac{PS}{10^{\frac{SNR}{10}}}$$

(4) $$ND=\frac{\sqrt{PN}}{\sqrt{\frac{\sum_{i=1}^{n}{\left(D-\mu \right)}^{2}}{n}}}*D$$

(5) NS=ND+x

In the above-listed formula, *d*_*i*_ was the randomly generated signal, *d*_*max*_ was the maximum value of the randomly generated noise signal, *x* was the original Raman spectral signal data, *n* was the number of random signals generated, and μ was the arithmetic mean of n random signals.

## Results

### Average Surface-Enhanced Raman Spectroscopy Spectra and Characteristic Peaks

**FIGURE 2:**
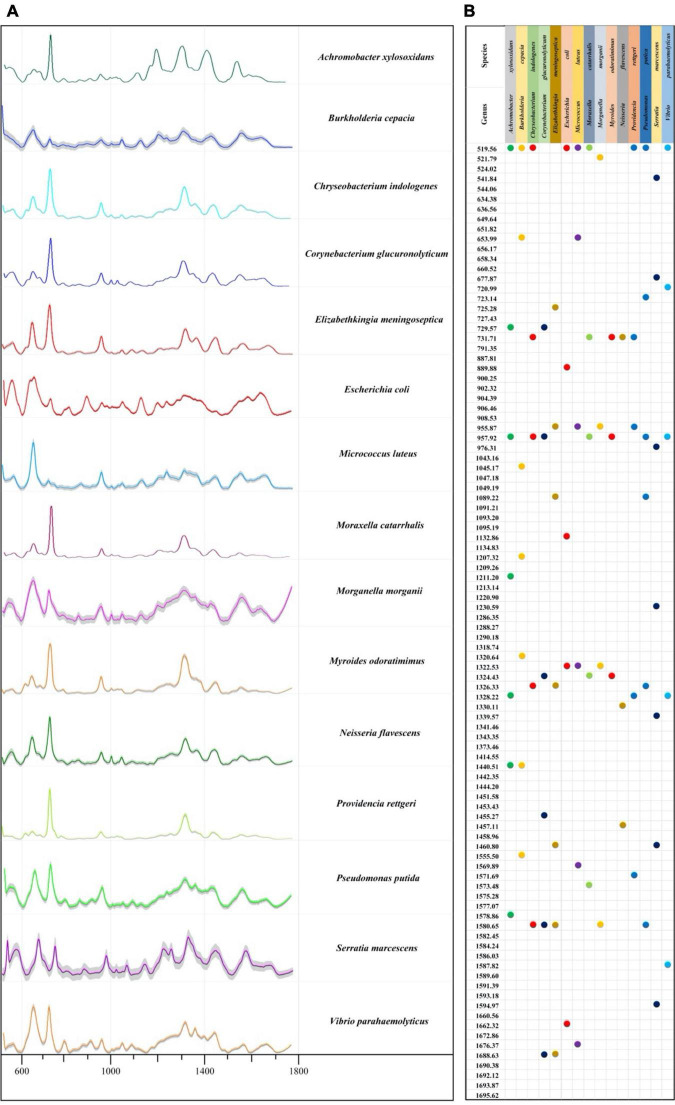
Average Raman spectra and the corresponding characteristic peaks of 15 bacterial pathogens isolated from clinical samples. **(A)** Average surfaced enhanced Raman spectra (SERS) spectra of 15 different clinical bacterial pathogens. Shaded part in each spectrum was 20% error band. **(B)** Dot plot distribution of characteristic peaks in the Raman spectra for 15 bacterial pathogens.

In this study, we calculated mean signal intensity at each Raman shift for a specific bacterial species to generate average SERS spectra, with the addition of 20% standard error (SE) band. The thinner the error band, the smaller the standard deviation, and the higher the reproducibility of Raman spectrum. After that, the software LabSpec was used to perform curve smoothing and denoising, baseline correction, and normalization operations on each average Raman spectrum. According to the results, all bacteria showed smooth and distinct spectral distributions with observable signal peaks at different Raman shifts (Figure 2A). Through analyzing average SERS spectra *via* the LabSpec software, it was shown that different bacterial pathogens had their own species-specific combinations of characteristic peaks (Figure 2B). In addition, according to previous studies, characteristic peaks of Raman spectra could be matched to metabolites (Tang et al., 2021; Wang et al., 2021). The corresponding metabolites of all the characteristic peaks in the SERS spectra of the 15 bacterial pathogens were found in literature and are presented in Supplementary Table 4.

### Clustering of Pathogenic Bacteria

**FIGURE 3:**
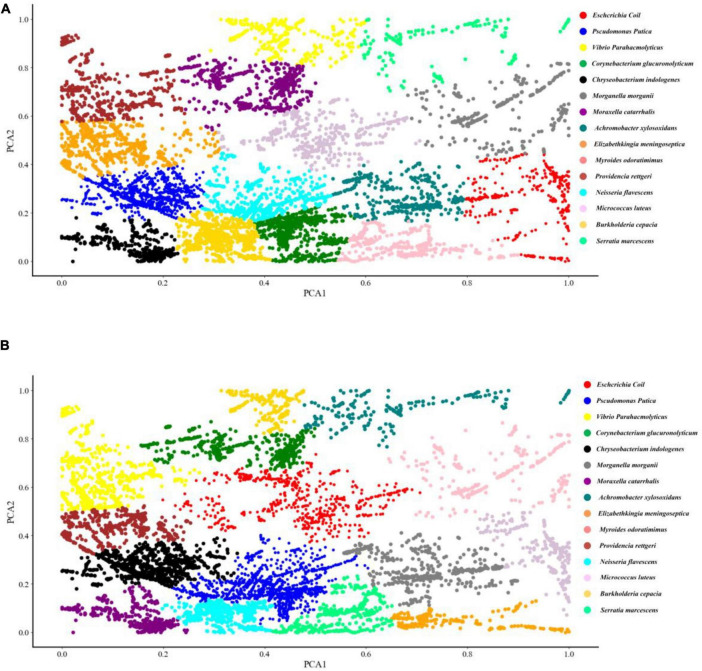
Schematic illustration of the clustering results of 15 bacterial pathogens used in this study *via* K-means and agglomerative nesting (AGNES). **(A)** K-means. **(B)** AGNES. Dots with different colors represented different bacterial pathogens as indicated by the figure legends on the right.

Two common unsupervised machine learning algorithms, K-means and AGNES, were used to classify the SERS spectral data into different groups. According to previous studies, K-means algorithm has already been successfully applied to analyse Raman spectra of biological samples. As for AGNES, it is a hierarchical clustering method that divides data into different sets through successive fusion of a single object, which has also been widely used in biological sample analysis. However, these two methods were rarely used for Raman spectral analysis. In this study, the clustering results of K-means and AGNES were shown in the form a scattering dot diagram in Figure 3. In order to quantify the clustering effects of the two methods, the metric Rand Index was used to evaluate the performance of the two algorithms. K-means algorithm achieved the highest score that was only 27.4%. The possible reason might be due to that data in the same Raman spectrum had large differences between the maximum and minimum intensities, and the number of samples of different bacterial pathogens were unevenly distributed, which made K-means unable to fit correctly.

### Prediction of Pathogenic Bacteria

Eight supervised machine learning algorithms were compared in this study to explore their predictive abilities in the identification of bacterial pathogens through the analysis of their SERS spectra, which included CNN, DT, GRU, LSTM, MLP, RF, SimpleRNN, and SVM. A total of four evaluation indicators, which were accuracy (ACC), precision (Pre), Recall and F1, together with 5-fold cross-validation were used to evaluate the performance of the eight algorithms. According to the results summarized in Table 1, CNN had the highest prediction accuracy (99.86%), and its five-fold cross-validation was the most robust with the overall accuracy of 99.47%.

**TABLE 1:** Comparative analysis of the predicative capabilities of eight machine learning algorithms on surfaced enhanced Raman spectra (SERS) spectral data belonging to 15 bacterial pathogens.

Algorithms	ACC	Pre	Recall	F1	5-Fold CV
CNN	99.86%	99.91%	99.91%	99.93%	99.47%
LSTM	98.87%	98.87%	92.20%	98.74%	96.76%
RF	98.71%	98.77%	98.80%	98.77%	98.35%
GRU	98.61%	97.91%	97.93%	97.92%	89.68%
SVM	97.30%	97.30%	97.08%	97.28%	97.93%
SimpleRNN	96.43%	96.91%	95.89%	95.91	83.63%
DT	96.01%	97.96%	97.53%	97.95%	97.48%
MLP	95.17%	96.07%	95.54%	95.86%	96.84%

**FIGURE 4:**
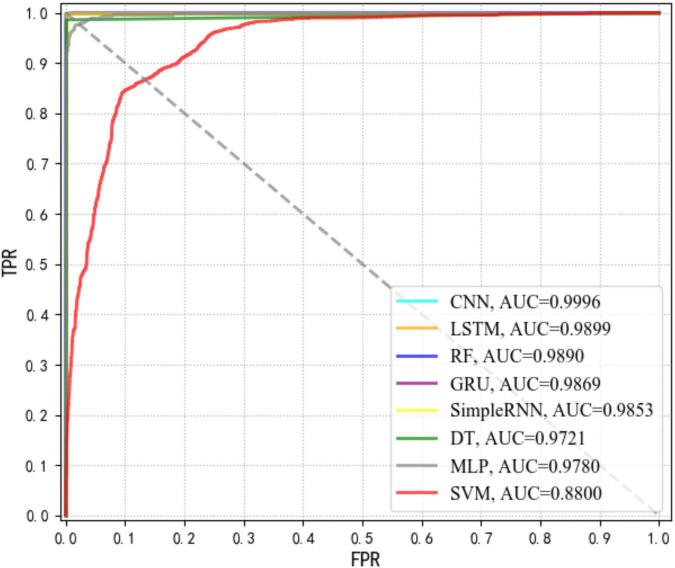
Comparison of receiver operating characteristic curve (ROC) curves *via* area under the curve (AUC) values for eight supervised machine learning algorithms.

In order to evaluate the diagnostic abilities of a supervised machine learning algorithm, a probability curve called receiver operating characteristic (ROC) curve was drawn at various threshold settings, through which sensitivities (true positive rate, TPR) and specificities (false positive rate, FPR) for different values of a continuous test were visualized (Hoo et al., 2017). In the ROC curve, upper left corner indicated higher TPR and lower FPR. Thus, regions in the ROC curves closer to the upper left corner had larger sum of sensitivity and specificity. In order to quantify TPR and FPR, the index area under the curve (AUC) was calculated, according to which, the larger the AUC value, the better the performance of a supervised machine learning model. According to the results in Figure 4, it was apparent that CNN had the highest AUC that was closely followed by LSTM.

**FIGURE 5:**
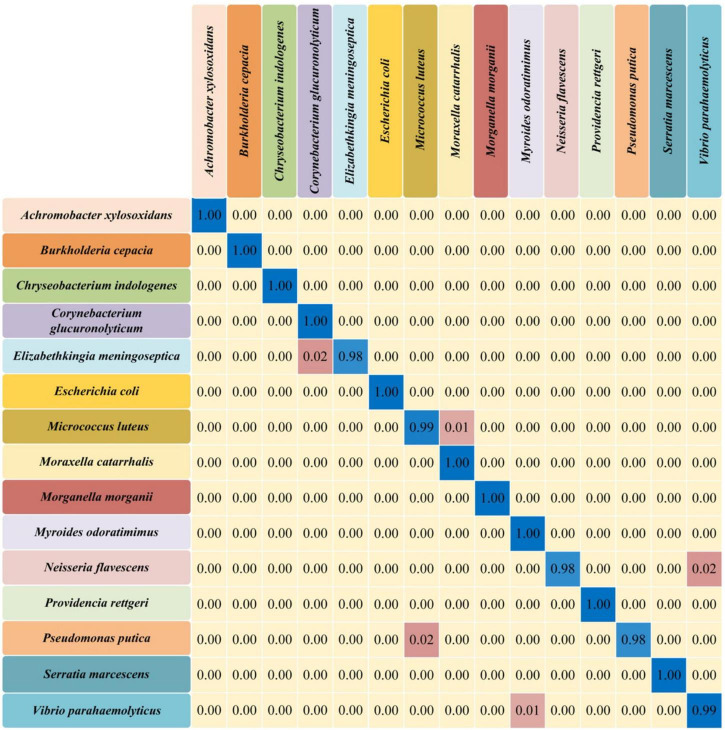
Confusion matrix of the convolutional neural network (CNN) model for 15 different bacterial pathogens. The rows in the confusion matrix represented the true categories of predictions, while the columns represented the categories of the incorrect predictions. The probability of correct prediction (diagonal) and the probability of incorrect prediction (off-diagonal) were all present in the matrix.

Confusion matrix is an evaluation table to quantify the classification performance of machine learning algorithms by using true class and predicted class. Each row of the matrix represents the probability that the model predicts a true sample, and each column represents the probability that the model predicts an incorrect sample. Since CNN model achieved the best performance in bacterial identification in this study, we calculated its confusion matrix to provide further classification details (Figure 5). According to the matrix, most of the bacterial pathogens could be accurately discriminated by the CNN model with 100% accuracy.

### Robustness of Machine Learning Methods

**FIGURE 6:**
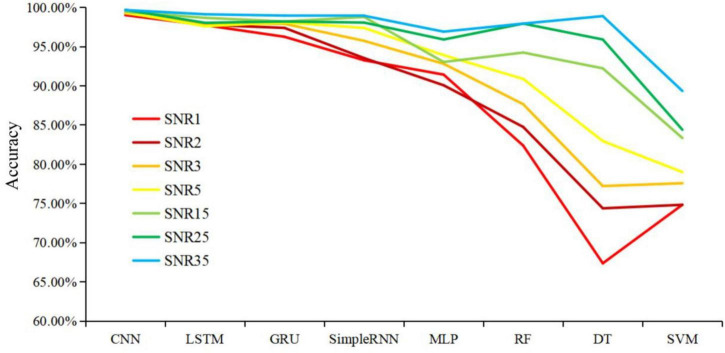
Schematic illustration of the influences of different signal-to-noise ratio on the prediction accuracies of eight machine learning algorithms. X-axis shows different machine learning models. Y-axis represents prediction accuracy of machine learning algorithms under different signal-to-noise ratios. Lines with different colors represent noises intensities. The smaller the signal-to-noise ratio (SNR) value, the more noise added to the spectra and the worse the data quality.

Noise sources could greatly compromise the quality of Raman spectra, causing issues in computational analysis of the spectral data and leading to inaccurate determination of specimen composition (Tuchin et al., 2017). However, it is not known to what degree that noises could influence the performance of machine learning algorithms. In order to check the impacts of noises on Raman spectral analysis, artificial noise intensity was added to the pre-processed SERS spectra at 1, 2, 3, 5, 15, 25, and 35 dB, respectively. Eight different machine learning algorithms were used to analyze the modified spectra, and the effects of noises on these models were assessed *via* prediction accuracy. According to the results shown in Figure 6, CNN maintained a consistently high and stable prediction accuracy at different noise intensities, which was followed by LSTM and GRU. The prediction accuracies of three algorithms were kept above 95%, showing good and stable performance during spectral data analysis. It could also be seen that the prediction accuracies of RF and DT models were less than 75% when SNR was equal to 1 or 2, which indicated that performance robustness of RF and DT models were poor with low SNRs.

## Discussion

Traditional methods for the detection of bacterial pathogens mainly rely on culture and biochemical tests to perform bacterial discrimination and phenotypic profiling, which, in spite of their high accuracies, are generally time-consuming and laborious while advanced molecular methods such as PCR and ELISA require specially designed primers or antibodies and have relatively high costs and/or false positive rates (Wang et al., 2021). For the newly developed high-throughput sequencing technology, although the cost of sequencing has dropped significantly, complex sample preparation procedures and data analysis processes have limited its wide application in clinical laboratories for routine diagnosis of bacterial pathogens (Deurenberg et al., 2017). As a sensitive, low-cost, label-free, and non-destructive technology of biological sample analysis, Raman spectroscopy has potential in promoting fast diagnosis of bacterial pathogens, though it is still considered as a novel technology and is too arbitrary to ascertain that RS can be applied in clinical settings at any time soon because a gap between basic research and clinical implementation still exists for the methodology (Wang et al., 2021).

Although SERS spectra have higher signal intensity and data quality than Raman spectra, they still need to be preprocessed in order to improve the performance of computational analysis (Xiong and Ye, 2014; Tang et al., 2021). In addition, due to the complexity of Raman spectral data, the classical linear method is no longer suitable for its spectral data analysis (Lussier et al., 2020). In this study, we aimed to understand the intrinsic differences among Raman spectra belonging to 15 bacterial pathogens through comparing the classification and prediction abilities of both unsupervised and supervised machine learning algorithms. Previously, PCA combined with HCA was successfully applied to cluster *Staphylococcus aureus* and *E. coli* into different groups (Boardman et al., 2016). In addition, Weng et al. (2018) applied K-means to urine samples for automatic filter of dynamic spectra and rapid detection of drugs in urine, while Geng et al. (2021) used hierarchical clustering analysis (HCA) to differentiate neural stem cells accurately through label-free Raman spectroscopy. In this study, we used two clustering algorithms, KMeans and AGNES, to classify the 15 bacterial pathogens. Pathogenic bacteria were clustered in Figure 3. However, due to the uneven distribution of the spectral data of different bacterial pathogens and the complex SERS spectral data, the clustering effects were not ideal, indicating that more advanced calculation methods were needed for further investigations. In addition to this, more data corresponding to distinct isolates and a more even representation of isolate variability within each species are issues that should be addressed in future research.

As for the prediction of bacterial species, we used three traditional machine learning algorithms (RF, DT and SVM) and five deep learning algorithms (CNN, GRU, LSTM, MLP, and SimpleRNN) to analyze SERS spectral data. Although many supervised machine learning algorithms were applied to the analysis of Raman spectra (Riva et al., 2021), few studies systematically compared the classification and prediction of multiple algorithms among clinical pathogens belonging to different genera. Previously, Tang et al. compared 10 supervised machine learning algorithms in terms of performance on 2,752 SERS spectra from 117 *Staphylococcus* strains belonging to nine clinically important *Staphylococcus* species, according to which all supervised machine learning models achieved good prediction results while CNN topped all other models and accurately predicted *Staphylococcus* species with the highest accuracy at 98.21% (Tang et al., 2021). In this study, our results suggested that the deep learning algorithm CNN had the best performance on SERS spectra (accuracy = 99.86%) for the prediction of bacterial species at general level (Table 1).

When dealing with low-dimensional data in small volumes, it is convenient to pick-up outlier values through inter-quartile range (IQR) analysis. However, the method is time-consuming and labor-intensive when applied to large-scale data and is not suitable for high-dimensional data analysis. Common methods for processing high-dimensional data include Mahalanobis distance, robust Mahalanobis distance and principal component measurement method (PCout). In particular, the Mahalanobis distance method could evaluate whether a spectrum is an outlier or not by comparing the distances of all the corresponding points between the tested spectra and all other spectra one by one (De Maesschalck et al., 2000). However, the method is not robust because individual outliers will cause the mean vector and covariance matrix to shift toward wrong direction, leading to abnormal Mahalanobis distance and misidentified outliers; in contrast, robust Mahalanobis distance method constructs a robust mean and covariance matrix estimator through iteration to identify outliers, which is able to solve the problem (Cabana et al., 2019). As the dimensionality increases, the distribution of data in the coordinate system will become increasingly sparse, leading to mis-judgement of real-data and increases and insufficiency of outlier identification through distance methods. Thus, in this study, we recruited PCount() function in the mvoutlier package of the R programming language for outlier identification and removal.

It should also be noted that SERS spectral preprocessing was important in reducing the noise signals and improving the predictive ability of the model. Noise in signals was unavoidable for Raman spectroscopy because of factors such as fluctuations of environmental conditions, sample contaminations, and background fluorescence, etc., leading to the generation of abnormal spectra data that compromised data quality (Xiong and Ye, 2014; Tuchin et al., 2017). Thus, in this study, artificial noises were added to SERS spectra to objectively evaluate the robustness of the model. According to the result in Figure 6, it was found that CNN maintained a consistently high and stable prediction accuracy at different noise intensities, indicating that the CNN had strong robustness in classification and prediction of different pathogens. Further research should focus on directly identifying bacterial pathogens from clinical samples such sputum, blood and urine, etc., which is very challenging and will greatly facilitate the application of Raman spectroscopy in the clinical settings.

## Conclusion

Raman spectroscopy has been widely investigated in terms of its capacities in rapid diagnosis of bacterial pathogens such as species discriminations, antibiotic resistance detections and toxin identifications, etc. However, there is no rationale to claim that Raman spectroscopy is applicable for microbiologists and clinicians in real-world situations because a wide gap still exists between basic research and clinical implementation. In this study, we used surface enhanced Raman spectroscopy combined with unsupervised and supervised machine learning algorithms to detect 15 bacterial pathogens sourced from clinical samples. According to the results, SERS could accurately identify bacterial pathogens at general level with comparatively high specificity and sensitivity through the assistance of machine learning methods. Comparative analyses of all the supervised machine learning algorithms used in this study revealed that the deep learning algorithm CNN had the best prediction performance. In addition, CNN also topped other algorithms in terms of robustness when dealing with SERS spectra with artificially added noises. However, there are still many machine learning algorithms that have not been explored and should be investigated in future studies. For example, when the sample datasets are limited, a generative adversarial network can be used to amplify data amount while for datasets with more Raman shifts and higher dimensions, wavelength selection could be used, which is conducive to identify and select important bands for down-stream analysis. Moreover, standardized Raman spectroscopy database with reproducible spectra for clinically important pathogens should also be constructed, which could greatly improve the implementation of Raman spectroscopy in clinical environments. Taken together, Raman spectroscopy is a promising technique with potential for label-free detection and non-invasive identification of clinical pathogens, which is worthy of extensive explorations in future studies.

## Data Availability Statement

The original contributions presented in the study are included in the article/Supplementary Material, further inquiries can be directed to the corresponding authors.

## Author Contributions

LW, XZ, and BG conceived and designed the experiments and provided the platform and resources. LW was responsible for project administration. J-WT, J-QL, X-CY, W-WX, Y-CP, and Q-HL carried out the experimental work. LW and J-WT performed the data analysis. LW, J-WT, and Q-HL wrote and revised the manuscript. All authors read and approved the final manuscript.

## Conflict of Interest

The authors declare that the research was conducted in the absence of any commercial or financial relationships that could be construed as a potential conflict of interest.

## Publisher’s Note

All claims expressed in this article are solely those of the authors and do not necessarily represent those of their affiliated organizations, or those of the publisher, the editors and the reviewers. Any product that may be evaluated in this article, or claim that may be made by its manufacturer, is not guaranteed or endorsed by the publisher.
