# Supplementary material for: Rapid Discrimination of Clinically Important Pathogens Through Machine Learning Analysis of Surface Enhanced Raman Spectra
Source: Front Microbiol. 2022 Apr 8;13:843417. doi: 10.3389/fmicb.2022.843417 (PMC9024395; doi:10.3389/fmicb.2022.843417)
Supplement: Supplementary file 1 [file Table_1.DOCX]

**Supplementary Table S1** Basic morphological and physiological information of 15 bacterial species in this study.

| **Bacterial Species** | **Gram staining** | **Morphology** | **Niche** | **Spore** | **Oxygen** | **Oxidase** | **Catalase** | **Mobility** | **Drug Resistance** | **Clinical Symptoms** |
| --- | --- | --- | --- | --- | --- | --- | --- | --- | --- | --- |
| *Achromobacter xylosoxidans* | Negative | Rod | Wet environment | No | Aerobic | + | + | Positive | MDR^#^ | Pneumonia Pharyngitis Peritonitis Bacteremia |
| *Burkholderia cepacia* | Negative | Rod | Aquatic environment, Soil | No | Aerobic | Variable | + | Positive | MDR | Pneumonia |
| *Chryseobacterium indologenes* | Negative | Rod | Freshwater, Soil, Marine fish, Human hosts | No | Aerobic | + | + | Negative | MDR | Bacteremia Pneumonia Meningitis Myositis Keratitis |
| *Corynebacterium glucuronolyticum* | Positive | Rod | Trogenital tract | No | Aerobic | + | + | Positive | MDR | Genitourinary tract infections |
| *Elizabethkingia meningoseptica* | Negative | Rod | Freshwater, Saltwater, Soil | No | Aerobic | + | + | Negative | MDR | Pneumonia  Endocarditis Bacteremia |
| *Escherichia coli* | Negative | Rod | Intestines | No | Aerobic/Anaerobic | + | + | Positive | MDR | Diarrhea |
| *Micrococcus luteus* | Positive | Coccus | Skin, Soil, Dust | No | Aerobic | + | + | Nagative | MDR | Pain, Blurred vision, Awareness of white or yellow spot on cornea |
| *Moraxella catarrhalis* | Negative | Curved, straight or bean-shaped | Mucous membranes of the respiratory tract of mammals | No | Aerobic | + | + | Negative | MDR | Headache, Pain in the maxillary or frontal area, Fever, and Cough |
| *Morganella morganii* | Negative | Rod | Wastewater,Soil | No | Facultative anaerobic | - | + | Positive | MDR | Sepsis, Abscess, Cellulitis |
| *Myroides odoratimimus* | Negative | Rod | soil and water | No | Aerobic | + | + | Negative | MDR | Urinary tract infection |
| *Neisseria flavescens* | Negative | Flat-shaped | Human oropharynx | No | Aerobic | + | + | Negative | MDR | Vallecular Cysts |
| *Providencia rettgeri* | Negative | Rod | Wounds, Urinary tract, Reptile faces, Human blood | No | Aerobic | - | + | Positive | MDR | GastroenteritisBacteremia |
| *Pseudomonas putica* | Negative | Rod | Soil and Water habitats | No | Anaerobic | - | + | Positive | MDR | Bacteremia |
| *Serratia marcescens* | Negative | Rod | Water,Soil,food | No | Anaerobic | - | + | Positive | MDR | Respiratory Infections  Endocarditis  Arthritis  Osteomyelitis |
| *Vibrio parahaemolyticus* | Negative | Rod | Seafoods | No | Facultative anaerobic | + | + | Positive | MDR | Diarrhea Headache  Nausea |

Note: #MDR-multi-drug resistance.
