# Supplementary material for: Rapid Discrimination of Clinically Important Pathogens Through Machine Learning Analysis of Surface Enhanced Raman Spectra
Source: Front Microbiol. 2022 Apr 8;13:843417. doi: 10.3389/fmicb.2022.843417 (PMC9024395; doi:10.3389/fmicb.2022.843417)
Supplement: Supplementary file 2 [file Table_2.DOCX]

**Supplementary Table S2** Comparison of the number of surface enhanced Raman spectra before and after the removal of outlier spectra.

| **Bacterial species** | **Number of surface enhanced Raman spectra**  **(Before outlier removal)** | **Number of surface enhanced Raman spectra**  **(After outlier removal)** |
| --- | --- | --- |
| *Achromobacter xylosoxidans* | 610 | 480 |
| *Burkholderia cepacia* | 600 | 350 |
| *Chryseobacterium indologenes* | 690 | 466 |
| *Corynebacterium glucuronolyticum* | 600 | 578 |
| *Elizabethkingia meningoseptica* | 601 | 462 |
| *Escherichia coli* | 38 | 28 |
| *Micrococcus luteus* | 601 | 509 |
| *Moraxella catarrhalis* | 600 | 389 |
| *Morganella morganii* | 130 | 105 |
| *Myroides odoratimimus* | 610 | 512 |
| *Neisseria flavescens* | 601 | 494 |
| *Providencia rettgeri* | 601 | 483 |
| *Pseudomonas putica* | 100 | 100 |
| *Serratia marcescens* | 569 | 362 |
| *Vibrio parahaemolyticus* | 600 | 485 |
