# Supplementary material for: Rapid Discrimination of Clinically Important Pathogens Through Machine Learning Analysis of Surface Enhanced Raman Spectra
Source: Front Microbiol. 2022 Apr 8;13:843417. doi: 10.3389/fmicb.2022.843417 (PMC9024395; doi:10.3389/fmicb.2022.843417)
Supplement: Supplementary file 3 [file Table_3.DOCX]

**Supplementary Table S3** Summary of optimized parameters for all the eight supervised machine learning algorithms used in the study.

| **Models** | **Tuning** | **Optimized tuning parameters** |
| --- | --- | --- |
| CNN | Con Layer | Layer=6 |
|  | Max Pooling Layer | Layer=3 |
|  | filter | 5*1, 3*1 |
|  | activation | tanh, softmax |
|  | loss | categorical_crossentropy |
|  | optimizer | adam |
| DT | criterion (standards used for splitting nodes) | criterion = "gini" |
|  | splitter (select the split strategy on each node) | splitter = "best" |
|  | Depth (maximum depth of number) | Depth = "None" |
|  | min_samples_split (minimum number of samples) | min_samples_split = 2 |
| GRU | GRU Layer | Layer=3 |
|  | Dropout Layer, Dropout rate | Layer=2, rate=0.2 |
|  | activation | relu, softmax |
|  | loss | categorical_crossentropy |
|  | optimizer | adam |
| LSTM | LSTM Layer | Layer=3 |
|  | Dropout Layer, Dropout rate | Layer=2, rate=0.2 |
|  | activation | relu, softmax |
|  | loss | categorical_crossentropy |
|  | optimizer | adam |
| MLP | Dense Layer | Layer=3 |
|  | Dropout Layer, Dropout rate | Layer=3, rate=0.1, 0.2 |
|  | activation | relu, softmax |
|  | loss | categorical_crossentropy |
|  | optimizer | adam |
| RF | Subtrees (# n_estimators) | n_estimators=100 |
| SimpleRNN | SimpleRNN Layer | Layer=3 |
|  | Dropout Layer, Dropout rate | Layer=2, rate=0.2 |
|  | activation | relu, softmax |
|  | loss | categorical_crossentropy |
|  | optimizer | adam |
| SVM | C (Penalty coefficient) | C=1 |
|  | kernel | kernel="rbf" |
|  | gamma (Kernel function coefficient) | gamma=0.001 |
|  | degree (Polynomial kernel order) | degree=3 |
|  | decision_function_shape | decision_function_shape='ovr' |
