# Supplementary material for: Rapid Discrimination of Clinically Important Pathogens Through Machine Learning Analysis of Surface Enhanced Raman Spectra
Source: Front Microbiol. 2022 Apr 8;13:843417. doi: 10.3389/fmicb.2022.843417 (PMC9024395; doi:10.3389/fmicb.2022.843417)
Supplement: Supplementary file 4 [file Table_4.docx]

**Supplementary Table S4** Band assignments of characteristic peaks to potential metabolites in the average Raman spectra of 15 bacterial pathogens analyzed in this study.

| **Raman Shift (**$\mathbf{cm}^{\mathbf{-1}}$**)** | **Band Assignment** | **Reference** |
| --- | --- | --- |
| 520, 522, 542 | S-S stretching | ^1^ |
| 654 | C-C | ^2^ |
| 678 | Guanine | ^3^ |
| 721-732 | Nucleic acids | ^4^ |
| 890 | Tryptophan | ^5^ |
| 956, 958 | C=C | ^6^ |
| 976 | Ribose | ^7^ |
| 1045 | C-O | ^8^ |
| 1090 | Phenylalanine | ^6^ |
| 1133 | C-N | ^9^ |
| 1207, 1211 | C=S | ^10^ |
| 1230 | Amide Ⅲ | ^11^ |
| 1320-1340 | Adenine ring | ^12^ |
| 1441-1461 | N=N aromatic and aliphatic | ^10^ |
| 1555-1573 | Aromatic chain | ^13^ |
| 1579-1595 | Guanine, Adenine | ^12^ |
| 1662-1689 | C=O, C=C | ^10^ |

Note: S-S stretching represents a disulfide bond and corresponds to protein secondary structural changes ^14^. S-S stretching vibrational mode was found in the SERS spectra of most of the bacterial pathogens in the Raman shifts 520, 522, and 542 cm^-1^ ^1^ except for *Corynebacterium glucuronolyticum*, *Neisseria flavescens*, *Myroides odoratimimus*, and *Elizabethkingia meningoseptica*. As for the C-C bond (654 cm^-1^), the characteristic peak was found in the bacteria *Burkholderia cepacia* and *Micrococcus luteus* ^2^ *while* guanine (678 cm^-1^) was only identified in the bacterium *Serratia marcescens* ^3^. For nucleic acids (721-732 cm^-1^), ten bacterial species had characteristic peaks in the Raman shift region while five bacterial species, *Escherichia coli*, *Burkholderia cepacia*, *Morganella morganii*, *Serratia marcescens* and *Micrococcus luteus,* did not have the characteristic peaks ^4^. *E. coli* had a unique characteristic peak at 890 cm^-1^, which indicated the presence of tryptophan ^5^, while *Serratia marcescens* had a distinctive peak at 976 cm^-1^ (ribose) ^7^. It was also observed that C=C double bond at the Raman shifts of 956 and 958 cm^-1^ that was present in 11 bacterial species ^6^. There were also several characteristic peaks that were scarcely distributed in bacterial pathogens, which included C-O at 1045 cm^-1^ (*Burkholderia cepacia*) ^8^, phenylalanine at 1090 cm^-1^ (*Corynebacterium glucuronolyticum*, *Elizabethkingia meningoseptica*) ^6^, C-N at 1133 cm^-1^ (*Escherichia coli*) ^9^, C=S at 1207 and 1211 cm^-1^ (*Achromobacter xylosoxidans*) ^10^, and amide III at 1230 cm^-1^ (*Serratia marcescens*) ^11^. As for adenine ring (1320-1340 cm^-1^) ^12^, all the 15 bacterial pathogens had characteristic peaks in the region. In addition, N=N aromatic and aliphatic (1441-1461 cm^-1^) ^10^, aromatic chain (1555-1573 cm^-1^) ^13^, guanine/adenine (1579-1595 cm^-1^) ^12^, and C=O/C=C (1662-1689 cm^-1^) ^10^ were also scarcely distributed in the SERS spectra of the 15 bacterial pathogens.

**References**

1 Ahmed, M., Almagedi, S. & Yao, W. SERS Signatures of foodborne pathogenic zoonotic bacteria using gold colloid. *International Journal of Engineering Science and Technology* **5**, 12 (2013).

2 Feng, S. *et al.* Surface-enhanced Raman spectroscopy of saliva proteins for the noninvasive differentiation of benign and malignant breast tumors. *International journal of nanomedicine* **10**, 537-547, doi:10.2147/IJN.S71811 (2015).

3 Kim, J., Park, H., Kim, J., Chang, B. & Park, H.-K. Label-free Detection for a DNA Methylation Assay Using Raman Spectroscopy. *Chinese Medical Journal* **130**, 1961, doi:10.4103/0366-6999.211874 (2017).

4 Chao, Y. & Zhang, T. Surface-enhanced Raman scattering (SERS) revealing chemical variation during biofilm formation: from initial attachment to mature biofilm. *Analytical and Bioanalytical Chemistry* **404**, 1465-1475, doi:10.1007/s00216-012-6225-y (2012).

5 De Luca, A. C., Dholakia, K. & Mazilu, M. Modulated Raman Spectroscopy for Enhanced Cancer Diagnosis at the Cellular Level. **15**, 13680-13704 (2015).

6 ALmagedi, M. A. S.

7 Perez-Guaita, D. *et al.* Multimodal vibrational imaging of cells. **91**, 46-58 (2017).

8 Zhang, X., Yin, H., Cooper, J. & Haswell, S. Characterization of cellular chemical dynamics using combined microfluidic and Raman techniques. *Analytical and bioanalytical chemistry* **390**, 833-840, doi:10.1007/s00216-007-1564-9 (2008).

9 Chaturvedi, D. *et al.* Different Phases of Breast Cancer Cells: Raman Study of Immortalized, Transformed, and Invasive Cells. *Biosensors* **6**, 57, doi:10.3390/bios6040057 (2016).

10 Nguyen, E. P. *et al.* Assessment of a Raman micro-spectroscopy/microfluidics unit using a model E. coli/glucose bio-system. *The 7th IEEE International Conference on Nano/Molecular Medicine and Engineering*, 157-162 (2013).

11 Risi, R. *et al.* *X-ray radiation-induced effects in human mammary epithelial cells investigated by Raman microspectroscopy*. Vol. 8427 (2012).

12 Chisanga, M., Muhamadali, H., Ellis, D. I. & Goodacre, R. Surface-Enhanced Raman Scattering (SERS) in Microbiology: Illumination and Enhancement of the Microbial World. *Applied Spectroscopy* **72**, 987-1000, doi:10.1177/0003702818764672 (2018).

13 Nguyen, E. P. *et al.* Assessment of a Raman micro-spectroscopy/microfluidics unit using a model E. coli/glucose bio-system. 157-162 (2013).

14 Wang, C.-H., Huang, C.-C., Lin, L.-L. & Chen, W. The effect of disulfide bonds on protein folding, unfolding, and misfolding investigated by FT-Raman spectroscopy. *Journal of Raman Spectroscopy* **47**, 940-947, doi:10.1002/jrs.4935 (2016).
